# Histone H3 Lysine 27 Methylation Asymmetry on Developmentally-Regulated Promoters Distinguish the First Two Lineages in Mouse Preimplantation Embryos

**DOI:** 10.1371/journal.pone.0009150

**Published:** 2010-02-10

**Authors:** John Arne Dahl, Andrew H. Reiner, Arne Klungland, Teruhiko Wakayama, Philippe Collas

**Affiliations:** 1 Institute of Basic Medical Sciences, University of Oslo and Norwegian Center for Stem Cell Research, Oslo, Norway; 2 Centre for Molecular Biology and Neuroscience, Institute of Medical Microbiology, Oslo University Hospital and University of Oslo, Oslo, Norway; 3 Center for Developmental Biology, RIKEN, Chuo-ku, Kobe, Japan; CNRS, France

## Abstract

First lineage specification in the mammalian embryo leads to formation of the inner cell mass (ICM) and trophectoderm (TE), which respectively give rise to embryonic and extraembryonic tissues. We show here that this first differentiation event is accompanied by asymmetric distribution of trimethylated histone H3 lysine 27 (H3K27me3) on promoters of signaling and developmentally-regulated genes in the mouse ICM and TE. A genome-wide survey of promoter occupancy by H3K4me3 and H3K27me3 indicates that both compartments harbor promoters enriched in either modification, and promoters co-enriched in trimethylated H3K4 and H3K27 linked to developmental and signaling functions. The majority of H3K4/K27me3 co-enriched promoters are distinct between the two lineages, primarily due to differences in the distribution of H3K27me3. Derivation of embryonic stem cells leads to significant losses and gains of H3K4/K27me3 co-enriched promoters relative to the ICM, with distinct contributions of (de)methylation events on K4 and K27. Our results show histone trimethylation asymmetry on promoters in the first two developmental lineages, and highlight an epigenetic skewing associated with embryonic stem cell derivation.

## Introduction

Embryo development is regulated by the acquisition of distinct programs of gene expression as cells differentiate. Blastomere compaction and polarization at the 8–16 cell stage in the mouse embryo define inner and outer cells and provide the first sign of lineage specification. Inner cells give rise to the inner cell mass (ICM) which differentiates into embryonic lineages, while outer cells give rise to the trophectoderm (TE) which gives rise extraembryonic tissues [1]. Transcriptional programs regulated by gradually exclusive Cdx2, Eomes and Elf5 expression in the trophectoderm and Oct4, Nanog and Sox2 expression in the ICM underline this first lineage specification [2], [3]. Embryonic and extraembryonic lineages display differences in DNA methylation, with the placenta being hypomethylated, a condition reflecting the hypomethylated state of the TE relative to the ICM [2], [4]. In addition, immunolabeling studies have shown that histone H3 lysine 27 trimethylation (H3K27me3), a histone modification commonly associated with transcriptionally repressed genes, is more abundant in the ICM than in the TE [5]. This asymmetry in DNA and H3 methylation patterns reflects distinct gene expression programs and is believed to be important for lineage commitment [2], [4], [5].

Similarly to the ICM from which they are derived, embryonic stem cells (ESCs) are pluripotent; however unlike ICM cells which differentiate, ESCs can self-renew without compromising pluripotency [6]. Mouse ESCs display similarities with ICM cells, cells of the epiblast of early post-implantation embryos and with primordial germ cells, and like ICM cells, they are heterogeneous in their pattern of protein and gene expression [7], [8]. Unlike ICM cells however, ESCs are adapted to culture; protein expression is also interchangeable among cells in a given ESC culture and is associated with dynamic changes in histone modifications [7]. Thus, ESCs are likely to epigenetically diverge from the ICM and display complex histone modification patterns.

Genome-wide maps of posttranslational histone modifications, DNA methylation, and Trithorax and Polycomb target genes have unraveled chromatin states of pluripotency in ESCs [9]–[16]. These studies show that whereas H3K4me3 marks many promoters including those of highly expressed genes, H3K27me3 is enriched on promoters of inactive or weakly expressed genes. Undifferentiated cells also contain chromatin domains co-enriched in H3K4me3 and H3K27me3, which encompass genes that are transcriptionally halted or expressed at low level [9], [10]. Upon differentiation, these genes undergo demethylation on H3K27 and retain H3K4me3 when activated, or retain H3K27me3 and lose trimethylation on H3K4 when shut down [10], [11]. Co-enrichment of H3K4me3 and H3K27me3 on promoters has thus been proposed to constitute a mark of priming for transcriptional activation in undifferentiated cells. A similar picture emerges for lineage-specification genes in hematopoietic and mesenchymal progenitor cells [17], [18].

Except for information on a handful of genes [19], [20], virtually nothing is known on the genomic distribution of post-translationally modified histones in preimplantation embryos. This is presumably due to a lack of suitable tools. Genome-scale studies of mammalian embryos have been hampered by a requirement for large cell numbers for chromatin immunoprecipitation (ChIP), a technique widely used to map histone modifications and protein binding on the genome *in vivo* [21]. Here, we applied our micro (µ)ChIP assay for small cell numbers [22], [23] to map promoter occupancy of trimethylated H3K4 and H3K27 in the ICM and TE, and assess the dynamics of these modifications after derivation of ESCs.

## Results

### Profiling of H3K4 and H3K27 Trimethylation on Promoters in the ICM and TE

**Figure 1:**
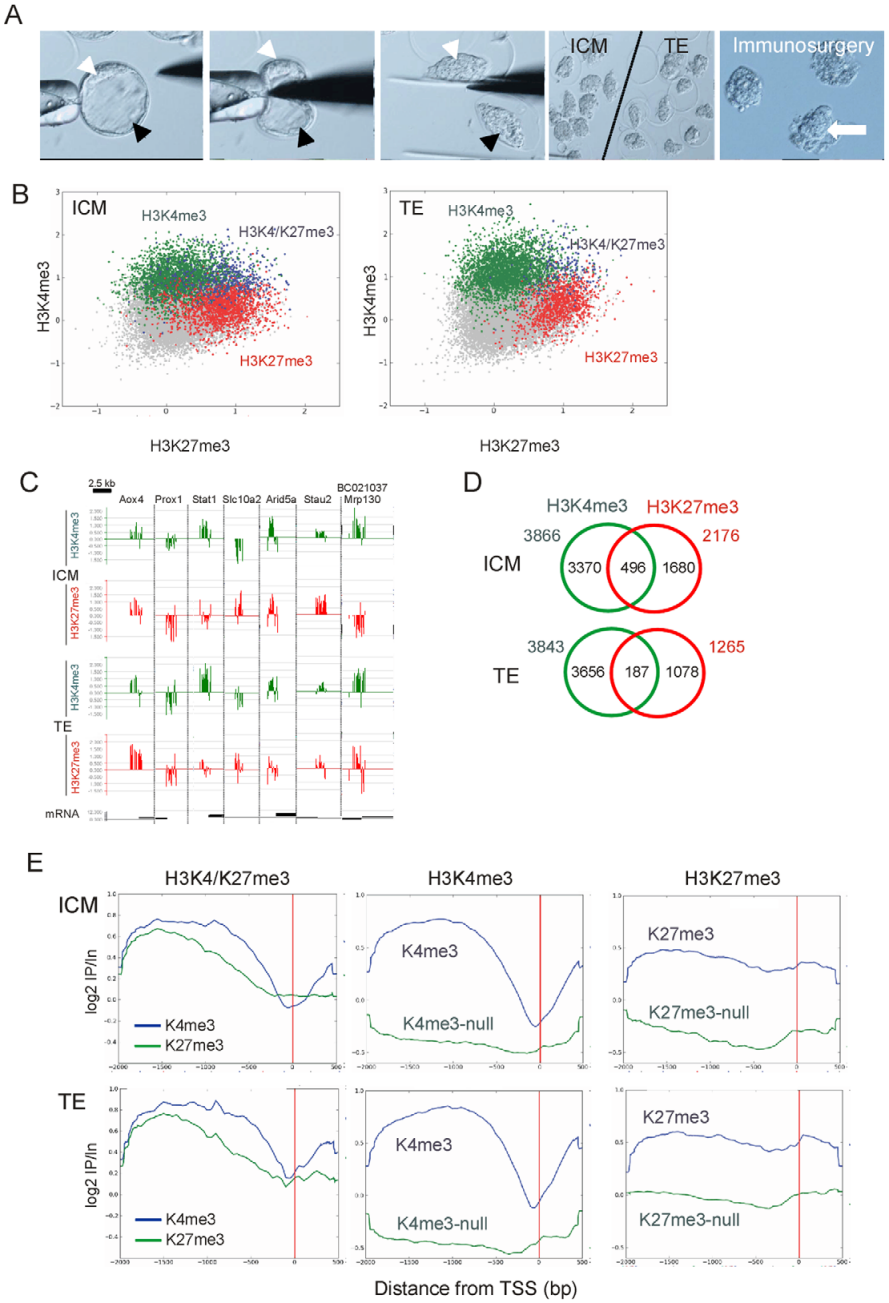
Distribution of H3K4me3, H3K27me3 and H3K4/K27me3 promoters in the ICM and TE. (A) Isolation of ICMs (white arrow) and TEs (black arrow) from E4 blastocysts by microdissection (left panels), followed by immunosurgery of the ICM/TE halves to purify the ICM (large arrow). (B) 2-D scatter plots of averaged MaxTen values for H3K4me3 vs. H3K27me3 log_2_ signal intensities in the ICM (left) and TE (right). Data points were colored to indicate classification according to the peak calling algorithm to show H3K4me3-enriched promoters (green), H3K27me3-enriched promoters (red) and promoters co-enriched in H3K4me3 and H3K27me3 (blue). (C) H3K4me3 and H3K27me3 enrichment profiles on indicated promoters in the ICM and TE. Data are expressed as log_2_ ChIP/Input ratios. (D) Venn diagram analysis of H3K4me3 and H3K27me3 promoters in ICM and TE. (E) Average distribution of H3K4me3 and H3K27me3 on H3K4/K27me3, H3K4me3 and H3K27me3 promoters, relative to the position of the TSS (red bar).

Mouse blastocysts cultured *in vitro* from the two-cell stage contain >60 cells, including ∼20 in the ICM and the rest in the TE. We purified TEs by bisection and ICMs by dissection followed by immunosurgery (Figure 1A). Isolated ICMs and TEs were viable because they reformed new blastocysts and trophoblastic vesicles, respectively (Figure S1). ICM and TE chromatin was subjected to triplicate H3K4me3 and H3K27me3 µChIPs and ChIP DNA was hybridized to microarrays tiling −2 to +0.5 kb relative to the transcription start site (TSS) of ∼27,000 promoters, including 19,489 RefSeq promoters. Reproducibility of µChIP-chip relative to Q^2^ChIP-chip (from 100,000 cells) and between µChIP-chip replicates has previously been reported [23]. Reproducibility was further shown here by two-dimensional scatter plots of MaxTen values for H3K4me3 and H3K27me3 (Figure S2A,B), and by the similarity of average enrichment profiles on metagenes (Figure S2C) and of promoter-specific enrichment patterns (Figure S2D and Figure S3).

Two-dimensional scatter plots of MaxTen values for H3K4me3 vs. H3K27me3 log_2_ signal intensities for all RefSeq promoters in the ICM and TE showed distinct enrichment patterns in each lineage (Figure 1B,C). Using a peak detection algorithm with a false discovery rate (FDR) of ≤0.1 for identification of enrichment in either modification, we showed that the ICM and TE contain a similar number of H3K4me3-enriched promoters, while the ICM harbors more promoters enriched in H3K27me3 than the TE (Figure 1D). We also identified in both lineages promoters co-enriched in H3K4me3 and H3K27me3 (referred to hereafter as H3K4/K27me3 promoters; Figure 1D, intersects). These made up <10% of all peak-containing promoters in the ICM and TE but were nevertheless 2.5 times more frequent in the ICM than in the TE. Thus, H3K4/K27me3 promoters constitute a minor yet significant proportion of promoters in both lineages. These results show a predominance of promoters enriched in H3K4me3 over H3K27me3 in both the ICM and TE; this observation was not unexpected as the correlation of H3K27me3 with promoters has been shown to be in general rather low in other cell types [15], [24]. Additionally, the ICM harbors more H3K27me3 promoters than the TE, corroborating on the promoter scale the global enrichment of H3K27me3 in the ICM reported by immunolabeling [5].

To determine the extent of overlap of H3K4me3 and H3K27me3 in the average H3K4/K27me3 promoter, we computed a metagene profile for each modification over the tiled regions (Figure 1E). In both the ICM and TE, H3K4me3 displayed a wide enrichment over −1,800 to −500 bp relative to the TSS, followed by a dip immediately upstream of the TSS, suggestive of displaced or unstable nucleosomes at the TSS [25], [26]. H3K27me3 overlapped largely with H3K4me3 but declined more gradually toward the TSS (Figure 1E, left panels). In concordance with expression status, the seemingly ‘nucleosome-depleted’ region delineated by the H3K4me3 profile in H3K4me3 promoters was more pronounced than that of H3K4/K27me3 promoters (Figure 1E). This suggests that H3K4me3 distribution is influenced by co-enrichment of H3K27me3.

**Figure 2:**
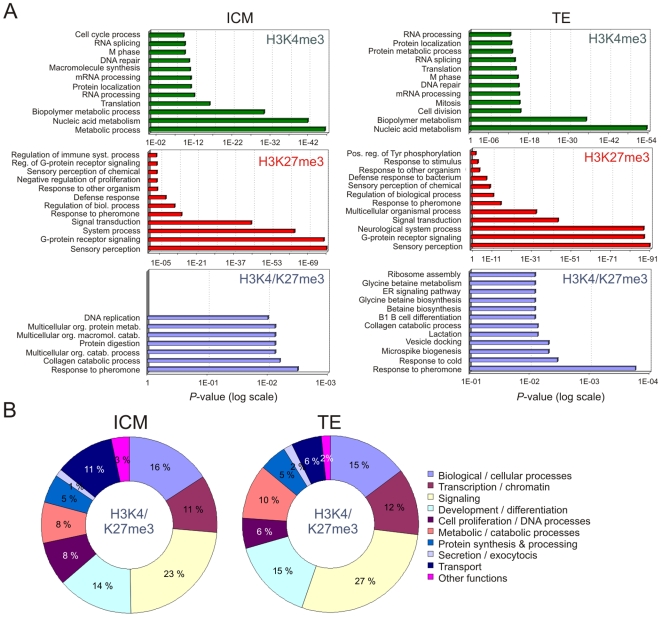
Genes with promoters enriched in H3K4me3 and/or H3K27me3 are associated with distinct functional categories. (A) GO term enrichment of genes containing H3K4me3, H3K27me3 or H3K4/K27me3 promoters in the ICM and TE. The twelve most significant GO terms are shown as a function of significance (*P*-value). (B) GO term representation of all genes containing H3K4/K27me3 promoters in the ICM and TE.

Gene ontology (GO) analysis indicated that in both the ICM and TE, H3K4me3 and H3K27me3 genes were enriched in housekeeping and signaling processes, respectively, wheras H3K4/K27me3 genes were predominantly linked to signaling, development/differentiation and transcription regulation functions (Figure 2A; Table S1). These functional categories were corroborated by the analysis of all GO terms identified among genes with promoters co-occupied by trimethylated H3K4 and H3K27 (Figure 2B; Table S2). These functional groups are remarkably similar to those reported in ESCs (see below), arguing that H3K4 and H3K27 trimethylation highlights similar sets of functions in embryonic cells, cultured or *in vivo*. Functional categories linked to H3K4 and H3K27 trimethylation are thus similar in the ICM and TE, although many genes carrying these modifications are distinct. Trimethylation of H3K4 and H3K27, therefore, delineates a cell identity profile in the ICM and TE.

### H3K4me3 and H3K27me3 Enrichment on Promoters of Genes Linked to Embryonic and Extraembryonic Development in the ICM and TE

**Figure 3:**
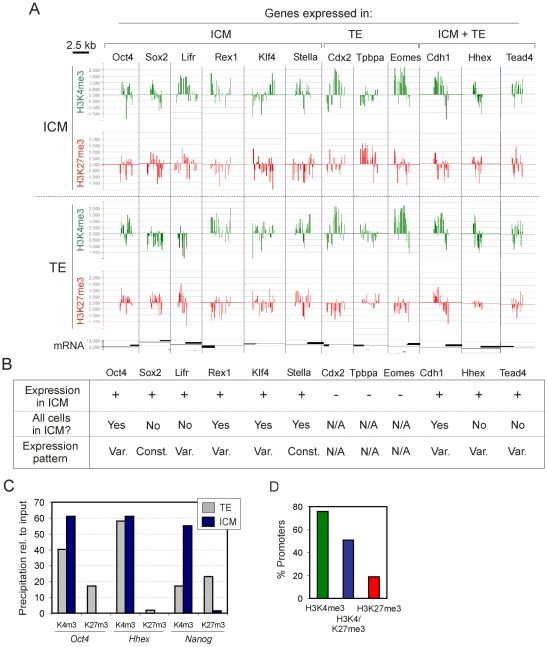
H3K4me3 and H3K27me3 enrichment profiles on genes expressed in the ICM and the TE. (A) µChIP-chip data of H3K4me3 and H3K27me3 enrichment profiles on promoters of indicated genes in the ICM and TE (log_2_ ChIP/input ratios). (B) Expression scoring and pattern of each gene examined in (A) in the ICM (*Var.*, variable expression level; *Const.*, consistent expression pattern). Data were extracted from published Affymetrix data [27]. (C) µChIP-qPCR analysis of H3K4me3 and H3K27me3 enrichment on the promoter of *Oct4*, *Nanog* and *Hhex* in the ICM and TE. (D) Percentage of expressed genes with promoters enriched in H3K4me3, H3K4/K27me3 or H3K27me3. Data were extracted from the Affymetrix dataset referred to in (B).

We next examined H3K4me3 and H3K27me3 profiles on promoters of genes reported to be expressed in the ICM and/or in the TE (Figure 3A,B) [1], [27]. Among genes expressed in the ICM, *Oct4*, *Sox2*, *Lifr*, *Rex1*, *Klf4* and *Stella* were either enriched in H3K4me3 relative to genome-average (*Oct4*, *Sox2*, *Lifr*, *Klf4*) or occupied by H3K4me3 at near genome-average level (*Rex1*, *Stella*). These promoters were either strongly hypo-trimethylated on H3K27 (*Sox2, Rex1, Klf4, Stella*) or harbored low levels of H3K27me3 (*Oct4, Lifr*). This was consistent with expression of these genes in the ICM, and notably with *Oct4* expression in a subpopulation of cells within the ICM [27]. In the TE, some of these genes were also enriched in H3K4me3 (*Oct4*, *Rex1*, *Klf4*, *Stella*) with enrichment in or low level H3K27me3, while others (*Sox2*, *Lifr*) harbored no H3K4me3 but were enriched in H3K27me3. These observations illustrate, therefore, similar H3K4 or H3K27 trimethylation profiles on a subset of genes (e.g., *Oct4*, *Rex1*, *Klf4* and *Stella*) in both the ICM and TE despite their distinct expression pattern in these compartments. Others, such as *Sox2* and *Lifr*, harbor H3K4me3 and H4K27me3 profiles that would be anticipated from their expression patterns.

Among genes expressed in the TE, *Cdx2*, *Tpbpa* and *Eomes* were occupied with (*Cdx2*, *Eomes*) or enriched in (*Tpbpa*) H3K27me3 in the ICM, and impoverished in H3K27me3 (*Cdx2*, *Eomes*) or occupied at genome-average level by H3K27me3 (*Tpbpa*) in the TE. These genes however showed no difference in H3K4me3 enrichment between the two compartments, consistent with observations that H3K4me3 can also occupy inactive promoters [28]. Lastly among genes expressed in both ICM and TE, *Hhex*, *Cdh1* and *Tead4* were enriched in H3K4me3 with little or no H3K27me3, suggesting mosaic expression in both lineages. µChIP-qPCR data corroborated µChIP-chip results and in addition showed that *Nanog* (not represented on the array) harbored H3K4me3 only in the ICM, and H3K27me3 with reduced H3K4me3 in the TE, as expected from its expression pattern in these compartments [1] (Figure 3C). We infer from these results that promoter enrichment in trimethylated H3K4 or H3K27 does not always correlate, in the embryo, with gene expression or repression, respectively (see also ref. [20]).

To examine this aspect further, we analyzed 15,941 cDNAs included in a published Affymetrix gene expression data set for twenty single ICM cells (GEO series GSE4307) [27]. Each probe on that array had a present/absent call and an expression index reported by DNA Chip Analyzer [29]. For each probe on the Affymetrix array, we derived a present/absent call by scoring ‘present’ if a signal was detected in ten or more of the twenty cell samples analyzed, in agreement with the method used to collapse replicates by DNA Chip Analyzer [29]. We found that in the ICM 76% of H3K4me3 promoters, 19% of H3K27me3 promoters and 51% of H3K4/K27me3 promoters were associated with expressed genes (Figure 3D). GO terms for all these expressed genes were linked to housekeeping functions regardless of H3K4 or H3K27 methylation state (Tables S3 and S4). These results imply that most H3K4/K27me3 genes encoding signaling and developmental functions in the ICM are in an inactive state. Nonetheless, detection of H3K27me3 on a subset of expressed genes in the ICM also suggests that these genes are not expressed in all cells of the ICM, in agreement with the mosaic expression of many genes in the ICM [1], [27]. Alternatively, some level of H3K27 trimethylation may be compatible with a transcriptionally active state [16].

### Trimethylation of H3K27 Is Asymmetrically Distributed between the ICM and the TE

**Figure 4:**
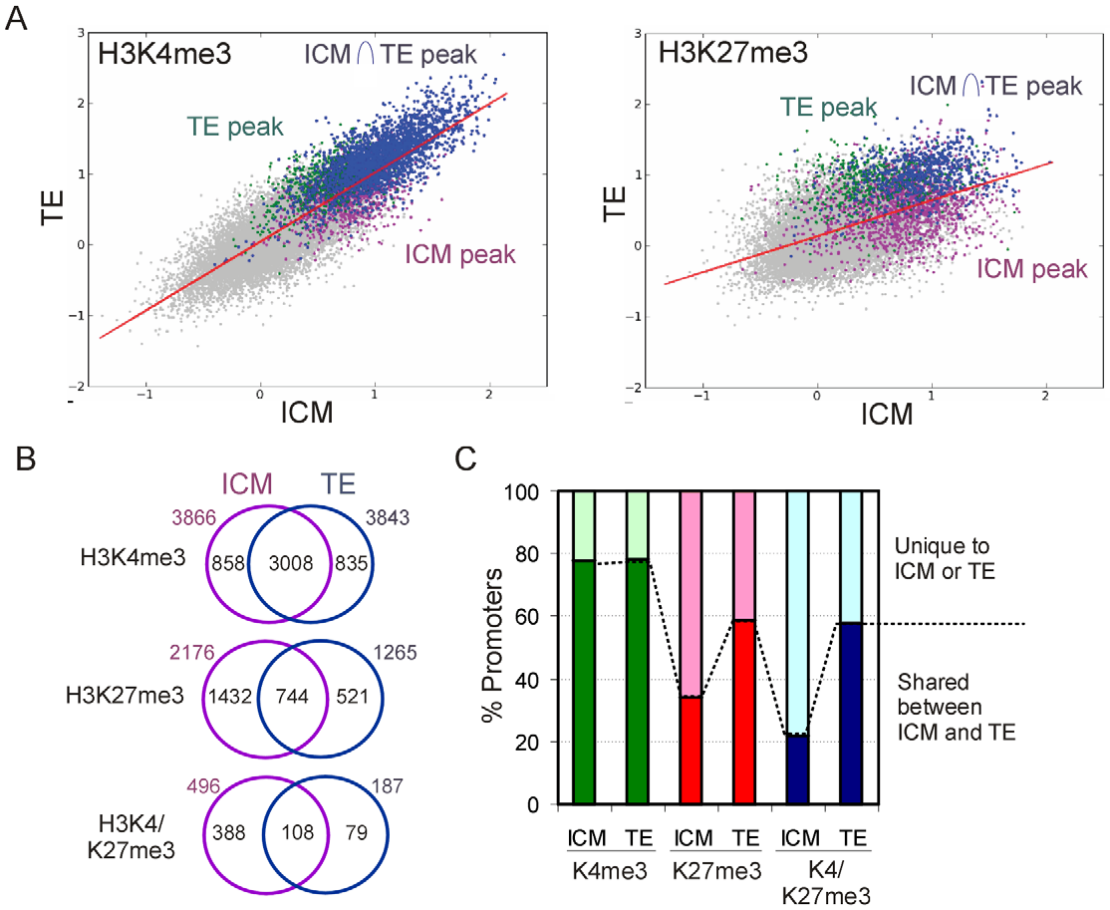
H3K27me3 is asymmetrically distributed in the ICM and TE. (A) 2-D scatter plots of averaged MaxTen values for H3K4me3 and H3K27me3 log_2_ signal intensities in ICM vs. TE. Data points were colored to indicate classification according to the peak calling algorithm to show H3K4me3- or H3K27me3-enriched promoters in all ChIP replicates in the TE (green), the ICM (purple) and common to both lineages (blue). (B) Venn diagram analysis of H3K4me3, H3K27me3 and H3K4/K27me3 promoters in ICM and TE. (C) Percentages of H3K4me3, H3K27me3 and H3K4/K27me3 promoters shared between ICM and TE, or unique to either lineage.

We next examined the extent of epigenetic overlap between the ICM and TE. Two-dimensional scatter plots of MaxTen values from H4K3me3 and H3K27me3 signal intensities in the ICM vs. TE (Figure 4A), together with peak identification (Figure 4B) showed greater overlap of H3K4me3 than H3K27me3 between the two lineages. Nearly 80% of H3K4me3 promoters in the ICM or TE were also enriched in H3K4me3 in the other compartment (Figure 4C). However, we found a lower proportion of H3K27me3 promoters (34%) and of H3K4/K27me3 promoters (22%) in the ICM that also contained these marks in the TE (Figure 4C). Therefore, in the blastocyst, H3K4me3 is largely conserved on promoters in both lineages, whereas there is significant asymmetry in the distribution of H3K27me3. This largely contributes to the asymmetry of H3K4/K27me3 promoter distribution between the ICM and TE.

In female mouse embryos, H3K27me3 has been shown to be enriched on the inactive X chromosome in the TE [5]. Interestingly, although the proportion of male and female embryos examined in our study was presumably equal, and although active and inactive X could not be distinguished on the array, we found in the TE a 3-fold enrichment of X-linked H3K27me3 promoters relative to the frequency of H3K27me3 promoters in the rest of the genome (data not shown). X-linked genes enriched in H3K27me3 in the TE are listed in Table S5.

### Dynamic Changes in H3K4 and H3K27 Methylation Patterns after Derivation of Embryonic Stem Cells

To identify H3K4 and H3K27 methylation changes associated with the establishment of ESCs, we derived ESCs from the ICM of B6D2F2 blastocysts (the strain examined here) and mapped H3K4me3 and H3K27me3 promoter enrichment profile. µChIP-chip from 1,000 ESCs notably revealed H3K4/H3K27me3 co-enrichment of the developmentally regulated *Hoxb* locus (Figure S4A) and enrichment of pluripotency genes in H3K4me3 with no or little H3K27me3 (Figure S4B). GO term enrichment analysis indicates that H3K4me3 enrichment was linked to housekeeping functions, whereas H3K27me3 and H3K4/K27me3 were associated with developmental and differentiation functions (Figure S4C; Table S6). These results validate µChIP-chip in relation to published data for ESCs [10], [11], [15], [16], [28]. Moreover, additional validation of our µChIP-chip approach was provided by cross-examination of H3K4me3- or H3K27me3 enriched genes identified in ESCs in our study with those identified by ChIP-sequencing in mouse ESCs [11]. This revealed that 76% and 56% of H3K4me3 and H3K27me3 genes, respectively, identified here were also found with these respective modifications by ChIP-sequencing [11] (data not shown). These proportions are consistent with overlaps between earlier published genome-wide investigations [11], [16], [28], [30].

**Figure 5:**
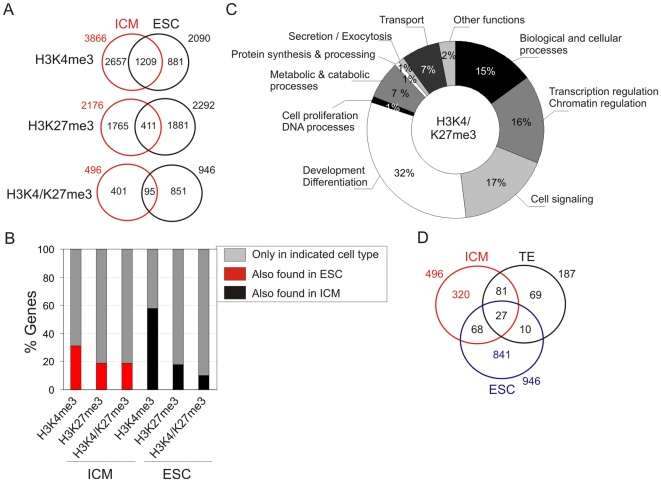
Divergence of H3K4me3 and H3K27me3 enrichment profiles between ICM and ESCs. (A) Venn diagram analysis of H3K4me3, H3K27me3 and H3K4/K27me3 promoters retaining and losing these marks after derivation of ESCs. (B) Percentages of promoters enriched in H3K4me3, H3K27me3 or both marks in the ICM and ESCs. (C) GO term representation of all H3K4/K27me3 genes identified in both the ICM and ESCs. (D) Venn diagram analysis of H3K4/K27me3 promoters in the ICM, TE and ESCs.

We next examined the extent of conservation of H3K4 and H3K27 trimethylation states between ICMs and ESCs (Figure 5A). Approximately 30% of H3K4me3 promoters and 20% of H3K27me3 or H3K4/K27me3 promoters in the ICM retained these marks in ESCs (Figure 5B, red bars). Thus most H3K4me3 and H3K27me3 promoters in the ICM lose trimethylation on K4 or K27, completely or to a level below peak detection threshold (Figure 5B, gray bars, left 3 columns). Similarly, ∼80% of H3K4/K27me3 promoters in the ICM harbored one or the other mark in ESCs by losing trimethylation on K4 or K27 or reducing levels thereof. In ESCs, over 40% of H3K4me3 promoters and over 80% of H3K27me3 and H3K4/K27me3 promoters gain these marks (Figure 5B, gray bars, right 3 columns). Consistent with our earlier findings, GO terms enriched and for all genes associated with common H3K4/K27me3 targets in the ICM and ESCs are involved in development and differentiation (32%), signal transduction (17%) and transcription regulation (16%) (Table S7; Figure 5C).

We infer from these observations that ESCs are epigenetically different from ICM cells. Loss of H3K4/K27me3 promoters upon derivation of ESCs does not result from an overt loss of methylation on one lysine or the other, but from a similar contribution of methylation loss on K4 and K27. In contrast, gain of trimethylation on H3K27 is more dynamic in ESCs than changes in H3K4 methylation. Further, the number of H3K4/K27me3 promoters in ESCs is greater than in the ICM, and the majority of these appear as ‘*de novo*’ H3K4/K27me3 promoters.

The different epigenetic patterns of ICM cells and ESCs prompted the determination of whether ICM and ES cells had more common bound targets than ICM and TE or TE and ES cells. Our data show that ICM and ESCs have a number of common H3K4/K27me3 targets (n = 105) similar to ICM and TE cells (n = 108); however, ESCs and TE cells share fewer common targets (n = 37, *P* = 0.013, Fisher's test; Figure 5D). ICM and ES cells seem therefore to be more closely epigenetically related to each another than ES and TE cells, an observation in line with the absence of similarity of mouse ESCs with cells of the TE [7]. There was nevertheless only minor overlap of targets between ICM, TE and ES cells (Figure 5D), indicating that these cell types are largely epigenetically distinct.

H3K4/K27me3 ICM-specific genes that lose methylation on either K4, K27 or both in ESCs, and those genes gaining H3K4/K27me3 in ESCs are listed with their GO terms in Table S8. We found for instance that H3K4/K27me3-marked telomerase (*Tert*) loses H3K27me3 to retain H3K4me3 in ESCs, consistent with expression of the gene in these cells. Similarly, several DNA repair genes (*Lig1, Poln, Gtf2h5*) also lose the H3K4/K27me3 double mark of the ICM to retain H3K4me3 in ESCs, suggesting an involvement of these genes in maintaining genomic integrity in rapidly dividing cells. We also found that genes involved in ICM proliferation, blastocyst development and implantation (*Luzp5, Surb7*, *Dnaja3*, *Grn*, *Egln1*) are marked by H3K4/K27me3 only in the ICM but not in the TE (Table S9), suggesting transcriptional posing of these genes by H3K27 trimethylation until their activation is required for development. Remarkably, all these genes lose methylation on both H3K4 and H3K27 in ESCs. Genes gaining H3K4/K27me3 in ESCs (Table S8) included the TE markers *Cdx2* and *Eomes*, and the *Hoxb* locus (implicated in anterior-posterior patterning), which is predominantly enriched in H3K4me3 only in the ICM. It emerges from these data that establishment of ESCs is associated with epigenetic changes reflecting a loss of function pertaining to early embryo development such as blastocyst development or patterning. These changes are paralleled by marked H3K4 and H3K27 methylation dynamics for a large number of genes implicated in several signal transduction pathways. These changes are likely to reflect the nature of functional changes taking place in ICM cells upon derivation of ESCs.

## Discussion

### Mapping of Histone Modifications in Preimplantation Mouse Embryos

We have interrogated by ChIP-on-chip sites of trimethylated H3K4 and H3K27 on RefSeq promoters in the ICM and TE, the first two distinct developmental lineages. This is to our knowledge the first genome-scale epigenetic profiling of preimplantation mammalian embryos, extending recent single gene-level studies [19], [20]. We reveal histone methylation asymmetry between the ICM and TE primarily caused by differences in promoter distribution of H3K27me3, and show that derivation of ESCs results in broad methylation changes on H3K4 and H3K27 relative to the ICM.

Co-enrichment and overlap of H3K4me3 and H3K27me3 on promoters suggests the existence of promoters harboring both marks in the embryo, adding to view that such promoters may exist in ESCs [9], [10], [16], progenitor cells [11], [15], [17], [18] and differentiated cells [24]. Overlapping of average profiles does not mean, however, that H3K4me3 and H3K27me3 peaks overlap on all promoters. Distribution of H3K4 and H3K27 methylation relative to each other [28] may impact on the activity of the target promoter. For instance, the depth of the H3K4me3 dip detected at the metagene level at the TSS on H3K4me3 promoters is not as pronounced among H3K4/K27me3 promoters. This suggests two subpopulations of such promoters: one with an H3K27me3 peak upstream of and non-overlapping the H3K4me3 peak, and one subpopulation with overlapping H3K4me3 and H3K27me3 peaks and no nucleosome-depleted region. On these promoters, the profile of H3K4me3 may be modulated by co-enrichment of repressive H3K27me3, which alters chromatin organization.

Co-detection of H3K4me3 and H3K27me3 in ICM, TE and ES cells may also reflect different epigenetic states in subpopulations of cells expressing different genes [7], [8], [27]. Heterogeneity in protein and gene expression is a hallmark of ESCs [7]. Remarkably, these expression states display a fluctuating equilibrium which parallels reversible histone modifications [7]. The ICM also contains a heterogeneous population of cells [1], exemplified by mutually exclusive expression of Nanog in the epiblast and Gata6 in the primitive endoderm [31]. Sorting expression profiles of single ICM cells [27] based on *Nanog* or *Gata6* mRNA levels indicates that even *Nanog*- or *Gata6*-expressing cells show variations in the nature of their transcripts [27]. These subpopulations therefore likely harbor different combinations of epigenetic marks on several loci.

Examples of trimethylated H3K4 and H3K27 profiles in the ICM and TE shown in Figure 3A are consistent with H3K4me3 enrichment or occupancy on most promoters irrespective of expression status [11], [28]. Moreover, extending recent carrier-ChIP data in cultured preimplantation mouse embryos [20], we find that there is not necessarily a robust correlation between H3K4me3 or H3K27me3 occupancy or enrichment and gene expression in the ICM or TE; this is particularly clear for H3K27me3. Accordingly, changes in gene expression during tissue regeneration cannot entirely be explained by these marks alone [32]. It has also been shown that differentiation can induce high levels of histone acetylation which may override the repressive effect of H3K27me3 [33].

Our data also infer that some genes may be regulated differently by, or independently of, occupancy levels of trimethylated H3K4 or H3K27 in the ICM and TE. To what extent these ‘inconsistencies’ reflect the *in vivo* situation in the embryo or *in vitro* embryo culture conditions remains to be examined. An additional component adding to the complexity of interpretation of H3K27me3 enrichment in particular is the newly discovered role of the H3K27me3 histone methyltransferase Ezh2 as a transcriptional activator on cell cycle- genes in cancer cells [34]. It is therefore possible that detection of H3K27me3 on specific loci may have a function other than transcriptional repression. One should also mention that a log_2_ ChIP/input probe signal of zero or above on arrays indicates some occupancy even tough there might not be enrichment relative to genome average (see Figure 3A). Such signals would be detected as positive by ChIP-qPCR, commonly expressed as a percent of input DNA. Negative levels are in contrast indicative, in the present study, of hypomethylation.

### Trimethylation of H3K4 and H3K27 Delineates a Cell Identity Profile in the ICM and TE

We have identified nearly three times as many H3K4/K27me3 promoters in the ICM as in the TE, suggesting that transcriptional silencing mediated by Polycomb-mediated K27 trimethylation [35] is more widespread in the ICM. This is in line with the greater abundance of Polycomb proteins in the ICM than TE [5], and with a requirement for the activation of a greater number of genes to initiate embryonic development than placental differentiation. H3K4me3 and H3K27me3 profiles in the ICM and TE may thus reflect the developmental fate of these lineages. Although the function of genes associated with H3K4/K27me3 promoters largely overlaps between ICM, TE and ESCs, there is only partial overlap of the target genes themselves. Thus each of these cell types retains some identity despite all being undifferentiated, a function suggested to be linked to the H3K27 methylase activity of Ezh2 [36]. The distinct developmental fates of the ICM and TE may speculatively be provided by the association of Ezh2 with different repressor complexes in these lineages [37], which have at least some non-redundant target genes. This possibility remains to be investigated.

In the blastocyst, different types of chromatin marks are related to the function of the associated genes rather than to a particular cell type. In the ICM and TE, as in ESCs and progenitor cells [10], [16], [17], [28], [38], [39], H3K4me3 promoters belong to genes with housekeeping functions, most of which are expressed. In contrast, expression is attenuated by H3K27 methylation (82% of ICM H3K27me3 genes are repressed), which targets signaling and developmental genes. We found that ∼50% of H3K4/K27me3 genes in the ICM are expressed, a figure higher than the few percents of active H3K4/K27me3 genes in ESCs [11]. Thus H3K4/K27me3 co-enrichment in undifferentiated cells *in vivo* is as likely to mark active genes as repressed genes.

One of the earliest markers of epigenetic asymmetry between the ICM and the TE is DNA hypermethylation in the ICM [2], [4], [40]. DNA hypermethylation in the ICM parallels enrichment of H3K27me3 in this compartment [5], suggesting that these modifications contribute to the repression of developmentally-regulated genes. A role of DNA methylation in maintaining the embryonic lineage was shown to depend on methylation of the TE-specific factor Elf5, whose expression is regulated by DNA methylation, and which positively regulates *Cdx2* and *Eomes* expression [2]. Of note, we found that *Elf5* is only trimethylated on H3K4 in the TE, a state compatible with unmethylated DNA and active transcription, whereas it harbors neither H3K4me3 nor H3K27me3 in the ICM (where it is repressed), and in ESCs is methylated only on H3K27 (data not shown), in addition to being DNA methylated [2]. These observations highlight a role of DNA methylation and histone modifications in regulation of lineage-specific gene expression, and in maintenance of lineage determination in the embryo.

### Dynamic Changes in H3K4 and H3K27 Methylation Contribute to the ES Cell Epigenome

We show that epigenetic skewing takes place after derivation of ESCs, indicating that ESCs are epigenetically distinct from the ICM. This is evidenced by loss of, or reduction in, trimethylation on H3K4 and H3K27 from the ICM on most promoters. Because many cell divisions occur during ESC derivation, we cannot at present invoke the role of an active demethylation process over a lack of replication-coupled maintenance histone methylation. However, there is also a gain of H3K4/K27me3 promoters in ESCs which involves *de novo* K27 methylation preferentially over K4 methylation. This greater dynamics of H3K27 methylation changes reflects its importance in the regulation of complex differentiation functions.

These observations raise the question of if ESCs are epigenetically different from ICM cells, then how can one account for their pluripotency *in vivo*? First, H3K4 and H3K27 methylation profiles on genes associated with pluripotency are comparable in the ICM and ESCs (this paper) [19]. Second, although ESCs can contribute to chimeras and support full development, not all ESC cultures are able to do so. This inefficiency may involve irreversible changes in the epigenetic program of the cultured cells. Nevertheless, chromatin states can be reversed through nuclear reprogramming. Thus, alterations in epigenetic modifications, as evidenced upon derivation of ESCs, may be reverted when the cells are placed in a new environment, such as in the ICM of host embryos. The predominance of H3K27 (relative to H3K4) methylation changes after ESC derivation and in the developing embryo, together with the association of H3K27me3-enriched genes with signaling and developmental functions, argues in favor H3K27 (de)methylation dynamics as an important component of the epigenetic plasticity of embryos and undifferentiated cells.

## Materials and Methods

### Embryos, Cells and Antibodies

B6D2F2 embryos were collected at the two-cell stage from superovulated and bred B6D2F1 (C57BL/6JxDBA/2) mice and cultured for 3 days (4.5 days post-coitum) to the blastocyst stage [41]. Animal maintenance and experimentation were conformed to the *Guide for Care and Use of Laboratory Animals* and were approved by the Institutional Committee for Laboratory Animal Experimentation at the RIKEN Kobe Institute. Females were superovulated with an injection of 5 IU equine chorionic gonadotropin and 5 IU human chorionic gonadotropin 48 h apart, bred to B6D2F1 males and sacrificed at 1.5 days post-coitum to collect embryos. TEs were isolated by bisection and ICMs purified by bisection and immunosurgery [42]. We collected in this manner 317 purified ICMs which were pooled, and 352 TEs which were also pooled. This provided chromatin for 6 ChIPs from ICMs, and 6 ChIPs from TEs. B6D2F2 ESCs were derived and cultured without feeders [41] to passage 8. ESCs were characterized previously [43] and shown to be germ-line competent (our unpublished data). Antibodies to H3K4me3 (cat# Ab8580) and H3K27me3 (cat# 05-851) were from Abcam (Cambridge, UK, http://www.abcam.com) and Upstate (Charlottesville, VA, http://www.upstate.com), respectively.

### Ethics Statement

Protocols for animal handling and treatment were reviewed and approved by the Institutional Committee for Laboratory Animal Experimentation at the RIKEN Kobe Institute.

### Chromatin Immunoprecipitation

µChIP was done as described [22] from isolated ICMs and TEs. In short, 2.4 µg antibody was coupled to 10 µl Dynabeads Protein A (Invitrogen, Oslo, Norway; http://www.invitrogen.com) in RIPA buffer (10 mM Tris-HCl pH 7.5, 140 mM NaCl, 1 mM EDTA, 1% Triton X-100, 0.1% SDS, 0.1% Na-deoxycholate). ICMs and TEs were cross-linked in PBS/20 mM Na butyrate containing 1% formaldehyde for 8 min and quenched with 125 mM glycine. Samples were frozen in liquid nitrogen and stored at −80°C. Lysis buffer (50 mM Tris-HCl, pH 8, 10 mM EDTA, 1% SDS, protease inhibitor cocktail, 1 mM PMSF, 20 mM butyrate) was added to ∼200 µl to each frozen sample before pooling using siliconized pipette tips. Lysed cells were sonicated for 3×30 sec on ice with 30 sec pauses using a probe sonicator (Labsonic-M, 3-mm probe; cycle 0.5, 30% power; Sartorius AG, Goettingen, Germany, http://www.sartorius.com) to produce ∼400–500 bp chromatin fragments. Fragment size was assessed by quantitative (q)PCR as recently described [23]. RIPA buffer (300 µl, with protease inhibitors, 1 mM PMSF, 20 mM butyrate) was added, samples centrifuged at 12,000 g and 450 µl supernatant was transferred into a 1.5 ml tube. Another 450 µl RIPA buffer was added to the sedimented lysate, centrifugation was repeated and 490 µl supernatant was pooled with the first one. RIPA buffer was added to the pooled supernatants to 1.22 ml, and 200 µl aliquots were transferred into six tubes containing antibody-coated beads.

Beads were released into chromatin and rotated at 40 rpm for 2 h at 4°C. ChIP material was washed three times in 100 µl RIPA and once in 100 µl TE buffer, and transferred into a new tube while in TE. Elution buffer and 5 µg RNase were added after removal of TE. Samples were incubated at 37°C for 20 min on a thermomixer. Proteinase K (1 µl at 20 µg/µl) was added and DNA elution, cross-link reversal and protein digestion were carried out for 2 h at 68°C on a thermomixer followed by a second extraction for 5 min; both supernatants were pooled. ChIP samples were made up to 490 µl in elution buffer without SDS. ChIP DNA was purified by phenol-chloroform isoamylalcohol extraction, ethanol-precipitated with 10 µl acrylamide carrier and dissolved in 10 µl MilliQ water.

ChIP and input DNA were amplified using the WGA4 GenomePlex Whole Genome Amplification Kit (Sigma-Aldrich, St. Louis, MO, http://www.sigma-aldrich.com) omitting lysis and DNA fragmentation steps. DNA was cleaned up (QIAquick kit, Qiagen, Valencia, CA, http://www.qiagen.com), purified and diluted to 250–500 ng/µl in MilliQ water.

To establish the specificity of H3K4me3 and H3K27me3 enrichment, duplicate total H3 ChIPs were performed using an anti-H3 antibody (Abcam; cat# ab1791) precipitating any modified form of H3. As anticipated from precipitation of a widely distributed core histone, ∼80% of input DNA was precipitated under these conditions and hybridization to promoter arrays showed low or no enrichment over genome-average, with only 470 peaks detected in both replicates (data not shown). Moreover, four negative control ChIPs with a pre-immune rabbit IgG (Millipore, cat# PP64B; http://www.millipore.com) only precipitated minute amounts of DNA; these were estimated by spectrophotometry to represent ∼0.3% of input DNA and were essentially not detectable by qPCR (data not shown).

### Microarray Hybridization and Data Analysis

ChIP and input DNA fragments were labeled with Cy5 and Cy3, respectively, and co-hybridized on Roche Nimblegen (Madison, WI, http://www.nimblegen.com) MM8 RefSeq Promoter arrays covering ∼27,000 promoters including 19,489 RefSeq promoters, ranging from −2,000 to +500 bp relative to the transcription start site (TSS). Data were analyzed using NimbleScan [23], [44] and custom-written software. Peaks were detected by searching for four or more probes with a signal above a cut-off value using a 500-bp sliding window. Cut-off values were a percentage of a hypothetical maximum defined as (mean + 6[standard deviation]). log_2_ ChIP/Input ratio data were randomized 20 times to evaluate the probability of false positives, and each peak was assigned a false discovery rate (FDR) score. Normalization and peak detection were performed by Nimblegen according to their published protocols. This process uses a cut-off range of 90% to 15%, with higher cut-offs corresponding to more stringent peak detection, reflected in the FDR calculation. H3K4me3- and H3K27me3 enrichment was identified based on detection of at least one peak at FDR≤0.1 in both replicates (N = 2) or in two of three replicates (N = 3).

For scoring promoters for correlation analysis we assigned an amplification value to each promoter by applying the Maxfour algorithm with a 10-probe window [45] (MaxTen). For each promoter, the corresponding probes log_2_ ratios were scanned in genome order with a 10-probe window. The highest 10-probe average was used as the amplification value for the promoter. Averaged MaxTen values from two ChIP replicates for each cell type are reported.

Assembly of a metagene of histone modification enrichment was performed [23] using genes with identified peaks. For metagene analysis, we identified ‘null-peak’ tiled regions as those having all probe ratios below the minimal cut-off value for peak detection. The microarray data have been deposited in a MIAME compliant database and are available in the NCBI GEO database under accession number GSE17387.

GO terms were either identified for all genes in a particular set, or GO term enrichments within a target gene set were calculated. We calculated GO term enrichments using the GOstats package [46]. GOstats identifies functional terms for selected genes and provides a significance of enrichment for a term by producing a *P*-value indicating the probability that the identified term is enriched to a greater extent among the target genes than would be expected by chance, based on the number of genes in the genome that belong to this term. GO term identifications for all genes were computed using GO mapping (ftp://ftp.ncbi.nih.gov/gene/DATA/gene2go.gz).

### Analysis of Gene Expression from Affymetrix Arrays

The 15,941 cDNAs included in a published Affymetrix expression data set for 20 single ICM cells (NCBI GEO GSE4307) [27] were analyzed for histone methylation patterns. For each probe on the Affymetrix array, we derived a present/absent call by scoring ‘present’ if a signal was detected in ten or more of the 20 samples analyzed, in agreement with the method used to collapse replicates by DNA Chip Analyzer [29]. Expression array data have been deposited in a MIAME compliant database and are available in the NCBI GEO database under accession number GSE17387.

### Quantitative PCR

ChIP DNA was also analyzed by duplicate qPCR as described[22] using the following primers: *Hhex*, (F) tcccccgttctagacagt, (R) agcctctggaacctgga; *Nanog*, (F) ctatcgccttgagccgttg, (R) aactcagtgtctagaaggaaagatca; *Pou5f1/Oct4*, (F) ctgtaaggacaggccgagag, (R) caggaggccttcattttcaa. Annealing temperature was 60°C for all primer pairs.

## Supporting Information

Figure S1 Isolated ICMs and TEs are viable. (A) Separation of ICMs and TEs by bisection, as also shown in Figure 1A. (B,C) Three hours after bisection, ICMs recavitate to form new blastocysts (B) and TEs recavitate to form trophoblastic vesicles (C).
(0.28 MB DOC)

Figure S2 Validation and reproducibility of µChIP-chip. (A) 2-D scatter plots of MaxTen values for H3K4me3 and H3K27me3 log_2_ signal intensities detected by Q^2^ChIP vs. µChIP in mouse P19 embryonal carcinoma cells. Correlation coefficient (R) and regression line are shown. (B) 2-D scatter plots of MaxTen values for H3K4me3 replicate and H3K27me3 replicate log_2_ signal intensities detected by Q^2^ChIP (top graphs) and µChIP (bottom graphs). (C) Average H3K4me3 and H3K27me3 enrichment profiles on promoters, detected by Q^2^ChIP and µChIP. TSS is represented by a red bar. (D) H3K4me3 and H3K27me3 profiles detected by Q^2^ChIP and µChIP on promoters through 520 kb of mouse chromosome 7. Data are expressed as log_2_ ChIP/input ratios. Position of transcripts is shown as black bars on the mRNA track.
(0.16 MB TIF)

Figure S3 Enrichment of H3K4me3 and H3K27me3 in the ICM and TE (two replicates each) expressed as log_2_ ChIP/input ratios (y axes) over 220 kb of chromosome 17.
(0.08 MB TIF)

Figure S4 µChIP-chip H3K4me3 and H3K27me3 enrichment profiles in ESCs. (A) Enrichment profile across the *Hoxb* locus. (B) Enrichment profiles on indicated promoters. (C) GO term enrichment of genes with a promoter enriched in indicated histone modifications.
(0.20 MB TIF)

Table S1 GO terms enriched for genes with promoters containing H3K4me3, H3K27me3, both marks or none of the marks in the ICM and TE (Excel file).
(0.07 MB XLS)

Table S2 GO terms associated with all H3K4/K27me3 promoter-containing genes identified in the ICM and TE (Excel file).
(0.08 MB XLS)

Table S3 GO terms associated with all expressed H3K4me3, H3K27me3 and H3K4/K27me3 promoter-containing genes in the ICM (Excel file).
(0.20 MB XLS)

Table S4 GO terms enriched for expressed H3K4me3, H3K27me3 and H3K4/K27me3 promoter-containing genes in the ICM (Excel file).
(0.03 MB XLS)

Table S5 X-linked genes enriched in H3K27me3 in the TE (Excel file).
(0.02 MB XLS)

Table S6 GO terms enriched for genes with promoters containing H3K4me3, H3K27me3, both marks, or none of these marks in ESCs (Excel file).
(0.06 MB XLS)

Table S7 ICM vs. ESC comparison of GO terms enriched for genes with promoters containing H3K4me3, H3K27me3 or both marks (Excel file).
(0.12 MB XLS)

Table S8 GO terms and list of genes enriched in H3K4/K27me3 in the ICM only or in ESCs only (Excel file).
(0.29 MB XLS)

Table S9 GO terms and list of genes enriched in H3K4/K27me3 in the ICM only or in the TE only (Excel file).
(0.11 MB XLS)
