# Supplementary figures and images for: Histone H3 Lysine 27 Methylation Asymmetry on Developmentally-Regulated Promoters Distinguish the First Two Lineages in Mouse Preimplantation Embryos
Source: PLoS One. 2010 Feb 10;5(2):e9150. doi: 10.1371/journal.pone.0009150 (PMC2818844; doi:10.1371/journal.pone.0009150)

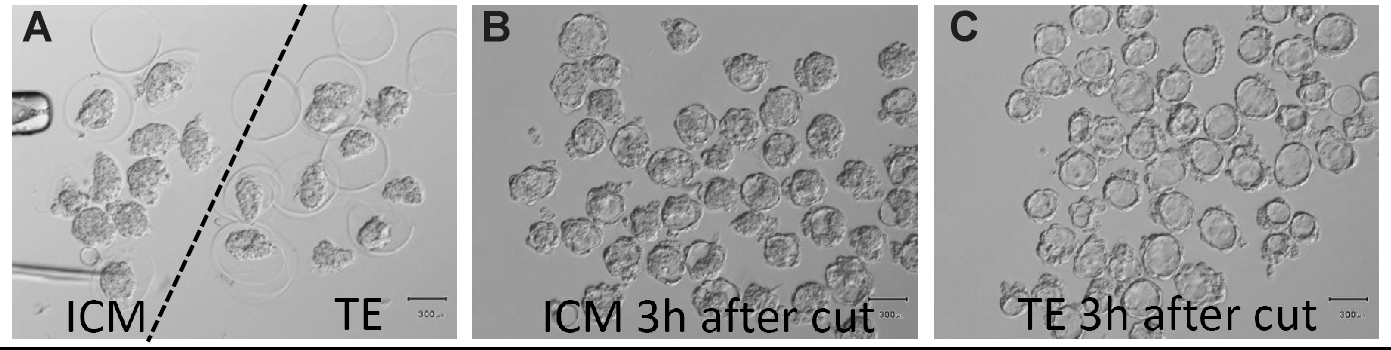

Supplement: Figure S1 — Isolated ICMs and TEs are viable. (A) Separation of ICMs and TEs by bisection, as also shown in Figure 1A. (B,C) Three hours after bisection, ICMs recavitate to form new blastocysts (B) and TEs recavitate to form trophoblastic vesicles (C). (0.28 MB DOC) [file pone.0009150.s001.tif]
